# Impaired Antibody-Dependent Cellular Cytotoxicity in a Spanish Cohort of Patients With COVID-19 Admitted to the ICU

**DOI:** 10.3389/fimmu.2021.742631

**Published:** 2021-09-20

**Authors:** Lorena Vigón, Javier García-Pérez, Sara Rodríguez-Mora, Montserrat Torres, Elena Mateos, María Castillo de la Osa, Miguel Cervero, Rosa Malo De Molina, Cristina Navarro, María Aránzazu Murciano-Antón, Valentín García-Gutiérrez, Vicente Planelles, José Alcamí, Mayte Pérez-Olmeda, Mayte Coiras, María Rosa López-Huertas

**Affiliations:** ^1^Immunopathology Unit, National Center of Microbiology, Instituto de Salud Carlos III, Majadahonda, Spain; ^2^AIDS Immunopathology Unit, National Center of Microbiology, Instituto de Salud Carlos III, Majadahonda, Spain; ^3^Serology Laboratory, National Center of Microbiology, Instituto de Salud Carlos III, Majadahonda, Spain; ^4^Internal Medicine Service, Hospital Universitario Severo Ochoa, Leganés, Spain; ^5^Neumology Service, Hospital Universitario Puerta de Hierro, Majadahonda, Spain; ^6^Neumology Service, Hospital de El Escorial, El Escorial, Spain; ^7^Family Medicine, Centro de Salud Doctor Pedro Laín Entralgo, Alcorcón, Spain; ^8^Hematology Service, Hospital Universitario Ramón y Cajal, Madrid, Spain; ^9^Division of Microbiology and Immunology, University of Utah School of Medicine, Salt Lake City, UT, United States

**Keywords:** COVID-19 severity, SARS-CoV-2 neutralizing antibodies, humoral response, antibody-dependent cellular cytotoxicity (ADCC), CMV reactivation, EBV reactivation

## Abstract

SARS-CoV-2 infection causes COVID-19, ranging from mild to critical disease in symptomatic subjects. It is essential to better understand the immunologic responses occurring in patients with the most severe outcomes. In this study, parameters related to the humoral immune response elicited against SARS-CoV-2 were analysed in 61 patients with different presentations of COVID-19 who were recruited in Hospitals and Primary Healthcare Centres in Madrid, Spain, during the first pandemic peak between April and June 2020. Subjects were allocated as mild patients without hospitalization, severe patients hospitalized or critical patients requiring ICU assistance. Critical patients showed significantly enhanced levels of B cells with memory and plasmablast phenotypes, as well as higher levels of antibodies against SARS-CoV-2 with neutralization ability, which were particularly increased in male gender. Despite all this, antibody-dependent cell-mediated cytotoxicity was defective in these individuals. Besides, patients with critical COVID-19 also showed increased IgG levels against herpesvirus such as CMV, EBV, HSV-1 and VZV, as well as detectable CMV and EBV viremia in plasma. Altogether, these results suggest an enhanced but ineffectual immune response in patients with critical COVID-19 that allowed latent herpesvirus reactivation. These findings should be considered during the clinical management of these patients due to the potential contribution to the most severe disease during SARS-CoV-2 infection.

## Introduction

Coronaviruses have become one of the main causes of emerged respiratory syndromes in the last decades (1, 2). By the end of 2019, the novel severe acute respiratory syndrome (SARS) coronavirus-2 (SARS-CoV-2), which is the causative agent of COVID-19, became responsible for the biggest pandemic in the recent History.

Clinical features of COVID-19 range from asymptomatic infection to critical or fatal disease (3), being the innate immune response and the cytokine storm considered the main causes for severity in the disease progression (4, 5). Accordingly, high levels of markers of inflammation in plasma such as interleukin (IL)-6, ferritin or C reactive protein (CRP) were described in patients with severe COVID-19 (5, 6). However, patients with extremely severe disease also showed low levels of cytokines with antiviral potential such as IL-2, IL-12 or interferon (IFN)-γ (7). Therefore, despite of the critical role of the inflammatory response during COVID-19, other specific immunological features that also contribute to the different clinical outcomes remain to be understood.

B lymphocytes are the immune adaptive cells responsible for the humoral response through the production of non-neutralizing and specific neutralizing antibodies, essential in the clearance of infections through different mechanisms such as opsonization and triggering of antibody-dependent cellular cytotoxicity (ADCC) (8). The generation of an stable humoral immune response requires the differentiation of both memory and plasma B cells (9). Naïve, antigen-specific B cells may proliferate and differentiate into plasmablasts, which are immature, short-lived antibody-secreting cells that eventually become plasma cells and produce large volumes of antibodies. These cells may become long-lived memory B cells if they maintain the B cell programming after the antigen wanes (10). A transient increase in circulating plasmablasts usually occurs during different viral and bacterial infections (11, 12). Accordingly, the level of plasmablasts also increases in peripheral blood during SARS-CoV-2 infection (13, 14). Furthermore, specific memory B cells against SARS-CoV-2 progressively increase during the first months of infection. These memory B cells could provide durable humoral immunity even if serum neutralizing antibody titers decline (15). However, the frequency and distribution of B cell subpopulations, defined by the expression of markers such as CD10, CD27 and CD21 (16), have not yet been extensively analyzed in patients with different presentations of COVID-19.

Neutralizing antibodies against SARS-CoV-2 structural proteins as nucleoprotein or spike may block viral infection of target cells. Higher titers of specific IgGs against SARS-CoV-2 are well-known to positively correlate with COVID-19 severity (15, 17–20). These antibodies potentially contribute to protect against SARS-CoV-2 infection not only by neutralization but also through the involvement of the complement system and ADCC by innate immune cells such as Natural Killer and macrophages. ADCC has been linked to the protection against many infectious diseases but also to the development of pathology (21). Therefore, a better understanding about how antibodies balance both protective and potentially pathogenic roles against SARS-CoV-2 is essential to take decisions about the clinical management of patients with COVID-19. So far, non-neutralizing antibody responses to SARS-CoV-2, including ADCC, are poorly understood although ADCC mediated killing is known to occur ex vivo in plasma from patients who have recovered from COVID-19 (22, 23).

On the other hand, progression of COVID-19 has been associated with the reactivation of herpesviruses such as Epstein-Barr virus (EBV), Varicella Zoster virus (VZV), Herpes Simplex virus type 1 (HSV-1) or cytomegalovirus (CMV), resulting in worse prognosis and even fatal outcomes that have been related to this reactivation (24–28). However, reactivation of herpesviruses has not been reported in large cohorts of patients with COVID-19.

In this study, parameters related to the humoral immune response that are elicited against SARS-CoV-2 in a Spanish cohort of patients with different COVID-19 presentations have been characterized, as well as the potential contribution to COVID-19 severe disease due to the reactivation of herpesviruses. These findings will improve our knowledge about the immune humoral response developed against COVID-19 and they could contribute to define new preventive measures during the clinical management of these patients.

## Methods

### Study Subjects and Samples

Blood samples were obtained from 61 patients with different presentations of COVID-19 who were recruited in Madrid (Spain) between April and June 2020, right after the first pandemic peak [estimated at the end of April (29)]. The inclusion criteria were to be over 18 year-old and to have a positive SARS-CoV-2 RT-qPCR assay in nasopharyngeal smear, which was performed in every hospital according to internal validated protocols. A total of 21 patients with mild COVID-19 required Primary Healthcare attention and were homebound until RT-qPCR assay for SARS-CoV-2 was negative; 40 patients required hospitalization and were further categorized as either severe (n=17) or critical (n=23) COVID-19. Critical patients required admission to the ICU. Clinical data from non-hospitalized and hospitalized patients are summarized in **Supplemental Tables 1** and **2**, respectively. Healthy donors (n=21) with similar age and gender distribution to mild COVID-19 patients were recruited as basal controls. Inclusion criteria for healthy donors were to be over 18 years old and have never been in contact with SARS-CoV-2 at the time of sampling.

### Cells

Peripheral blood mononuclear cells (PBMCs) and plasma were isolated from blood samples by centrifugation through Ficoll-Hypaque gradient (Pharmacia Corporation, North Peapack, NJ) and cryopreserved until the moment of analysis. Vero E6 (African green monkey kidney) cell line (ECACC 85020206) was kindly provided by Dr. Antonio Alcami (CBM Severo Ochoa, Madrid). Raji cell line (ATCC CCL-86) was provided by the existing cell collection of Instituto de Salud Carlos III (Madrid, Spain). Vero E6 and HEK-293T [National Institute for Biological Standards and Control (NIBSC)] cells were cultured in DMEM supplemented with 10% fetal calf serum (FCS), 2 mM L-glutamine and 100 units/ml penicillin and streptomycin (Lonza). PBMCs and Raji cells were cultured in RPMI supplemented with 10% FCS, 2 mM L-glutamine and 100 units/ml penicillin and streptomycin.

### SARS-CoV-2 Serology

IgG antibodies against SARS-CoV-2 spike protein were analyzed in plasma by direct enzyme-linked immunosorbent assay (ELISA) using Euroimmun Anti-SARS-CoV-2 Assay (Euroimmun, Lübeck, Germany), according to manufacturer’ instructions. Semi-quantitative results were analyzed by calculating the ratio of extinction of each plasma sample over the calibrator. The interpretation ratio was considered positive when IgG titer was >1.1; undetermined range was considered with values between 0.8 and 1.1; and values <0.8 was considered negative. Borderline data were considered positive.

### Pseudovirus Neutralization Assays

SARS-CoV-2 neutralization assays were performed using one single-cycle, pseudotyped virus encoding G614 SARS-CoV-2 spike (S) glycoprotein and *Renilla* luciferase gene, within the human immunodeficiency virus type 1 (HIV-1) genome (pNL4-3Δenv_ SARS-CoV-2-SΔ19(G614)_Ren). SARS-CoV-2 pseudotyped virus was prepared by co-transfection of HEK-293T cells with vector pNL4-3Δenv_Ren, which expressed HIV-1 genome without *env* gene and *Renilla* luciferase gene as reporter (30), together with vector pcDNA3.1-SARS-CoV-2-SΔ19, which expressed SARS-CoV-2 S glycoprotein (QHU36824.1), but lacking the last 19 amino acids (31), cloned into pcDNA3.1 expression vector (Invitrogen). Co-transfection with vector pcDNA-VSV-G, which expressed S glycoproteins of vesicular stomatitis virus (VSV), was used as control of specificity. The concentration of HIV-1 p24/Gag antigen in cell culture supernatants was quantified 48 hours post-transfection by electrochemiluminescence Immunoassay (Roche Diagnostic).

Neutralization activity was measured in plasma from COVID-19 patients by pre-incubation of 4-fold serial dilutions of heat-inactivated plasma (1/32 to 1/8192) with G614 SARS-CoV-2 pseudovirus (10ng p24 Gag per well) for 1 hour at 37°C, as previously described (32). Thereafter, this mixture was added to a monolayer of Vero E6 cells, and incubated for 48 hours. Vero E6 cells were then lysed and viral infectivity was assessed by measuring *Renilla* luciferase activity (Renilla Luciferase Assay, Promega, Madison, WI) using a 96-well plate luminometer Centro XS3 LB 960 with MikroWin 2010 software (Berthold Technologies, Baden-Württemberg, Germany). The titers of neutralizing antibodies were calculated as 50% inhibitory dose (ID50), expressed as the highest dilution of plasma that resulted in a 50% reduction of luciferase activity compared to control without serum, by non-linear regression using GraphPad Prism (GraphPad Software, Inc., San Diego, California).

### Herpesvirus Serology and Viral Reactivation

IgG antibodies against CMV, HSV-1, and VZV were analyzed in plasma from patients with COVID-19 using ELISA Enzygnost Anti-CMV/IgG, Anti-HSV/IgG, and Anti-VZV/IgG, respectively (Siemens Healthcare Diagnostics, Marburg, Germany). Results were expressed as differences in the optical densities (OD) between the viral antigen and control antigen. Therefore, ratio <0.100 was considered negative; ratio ≥0.100– ≤0.200 was undetermined; and ratio >0.200 was considered positive. IgG antibodies against EBV viral capsid antigens (VCA) were tested in plasma samples using the chemiluminescence immunoassay LIAISON VCA IgG assay (DiaSorin, Saluggia, Italy). Antibody titers were automatically calculated and expressed as units per milliliter (U/mL); the lower cut-off was 20 U/mL.

Viral reactivation of CMV and EBV in patients with COVID-19 was determined by extraction of total viral DNA from plasma using QIAamp MinElute Virus Spin Kit (Qiagen, Hilden, Germany). CMV and EBV genomic DNA was amplified and measured by qPCR in StepOne thermocycler (Applied Biosystems) using EBV R-GENE and CMV R-GENE kits (bioMérieux. Lyon, France), according to manufacturer’s instructions.

### B Cell Phenotyping

B cells are CD3^-^CD19^+^ cells with different phenotypes that may be determined by the analysis of the expression of surface markers CD10, CD27 and CD21 (16): immature or transitional cells (CD10^+^ CD27^-^); naïve B cells (CD10^-^CD27^-^CD21^high^); tissue-like memory cells (CD10^-^CD27^-^CD21^low^); resting memory cells (CD10^-^CD27^+^CD21^high^); activated memory cells (CD10^-^CD27^+^CD21^low^); and plasmablasts (CD27^++^CD20^-^CD21^low^) (16). Antibodies CD3-PE, CD10-BV421, CD19-BV711, CD20-AlexaFluor700, CD21-FITC, CD27-PercP-Cy5.5 were purchased from BD Biosciences (San Jose, CA). Data acquisition and analysis was performed as described above. The gating strategy for acquiring and analyzing the B cell subsets is described in **Supplemental Figure 1**.

### ADCC Assay

ADCC assay with PBMCs from patients with different presentations of COVID-19 was performed as described before (33) with some modifications. A total of 5x10^5^ Raji cells labeled with PKH67 Green Fluorescent Cell Linker (Merck KGaA, Darmstadt, Germany) were used as target cells. After staining, Raji cells were coated with rituximab (50µg/ml) (Selleckhem, Houston, TX) for 4 hours in the presence of 50% of pooled normal human serum (Innovative Research, Novi, MI). Labeled Raji cells were then co-cultured with PBMCs from COVID-19 patients (ratio 1:2) during 18 hours. Apoptosis of Raji cells was quantified by staining with Annexin V conjugated with 1.5 μM phycoerythrin (PE) (Immunostep, Salamanca, Spain), according to manufacturers’ instructions. Data acquisition was performed in a BD LSRFortessa X-20 flow cytometer using FACS Diva software (BD Biosciences, San Jose, CA). Data analysis was performed with FlowJo software (Tree Star Inc., Ashland, OR).

### Statistical Analysis

Statistical analysis was performed using GraphPad Prism. Statistical significance was calculated using ordinary one-way ANOVA and Tukey’s multiple comparisons test, with a single pooled variance. Correlations were calculated using Spearman correlation test. P values (*p*) < 0.05 were considered statistically significant in all comparisons and were represented as *, **, ***, or **** for *p*<0.05, *p*<0.01, *p*<0.001, or *p*<0.0001, respectively.

## Results

### Patients’ Cohorts

Sixty-one patients diagnosed with COVID-19 participated in this observational, cross-sectional study. Clinical data of patients are summarized in **Table 1**. Fifty percent of patients with mild COVID-19 were males, whereas males represented 70.6% and 60.8% of patients with severe and critical COVID-19, respectively. Median age of patients with mild COVID-19 was 42.0 years (interquartile range (IQR) 26 to 64) at the time of sampling, whereas median age of patients with severe and critical COVID-19 was 74.2 years (IQR 50 – 90) and 64.1 years (IQR 42 – 88), respectively. Due to the mandatory confinement decreed by the Spanish Government between March and June 2020, hospitalized patients with severe and critical COVID-19 were recruited in April and May, whereas homebound patients with mild COVID-19 were recruited once the confinement ended. Consequently, median number of days from clinical onset to sampling was 82.5 days (IQR 60 to 99) in mild COVID-19, 24.4 days (IQR 1-52) in severe COVID-19, and 34.8 days (IQR 7 to 59) in critical patients. Patients with mild COVID-19 did not develop pneumonia, but it was reported in 100% of patients with severe or critical COVID-19, respectively. Invasive mechanical ventilation was necessary in 5.8% of patients with severe COVID-19 and 73.9% of patients with critical COVID-19. Comorbidities such as diabetes, dyslipidemia, hypertension, and disseminated intravascular coagulation (DIC) were barely reported in patients with mild COVID-19 but they were in 23.5%, 70.5%, 64.7%, and 29.41% of severe patients and in 8.6%, 43.5%, 47.8% and 8.7% of critical patients, respectively. Exitus occurred in 7 patients with critical COVID-19 (30.4%). The most common symptoms in patients with mild COVID-19 were asthenia (85.0%), fever (65.0%), and cough (60.0%) (**Supplemental Table 1**). However, patients with severe and critical COVID-19 mostly presented with cough (52.9 and 69.5%, respectively), fever (35.3% and 73.93%, respectively) and dyspnea (47.0% and 69.5%, respectively) (**Supplemental Table 2**).

**Table 1:** Clinical data of patients with COVID-19 who participated in the study.

	Mild COVID-19	Severe COVID-19	Critical COVID-19
**Male/Female (n)**	10/11	12/5	14/9
**Age (years)**	42.2 (IQR 26 – 64)	74.2 (IQR 50 – 90)	64.1 (IQR 42 – 88)
**Days from clinical onset to sample**	82.5 (IQR 60-99)	24.4 (IQR 1-52)	34.8 (IQR 7-59)
**Days of hospitalization**	N/A	27.8 (IQR 8 – 67)	52.5 (IQR 2 – 110)
**Days of ICU stay**	N/A	N/A	40.4 (IQR 7 – 94)
**Pneumonia (Yes/No/UD)**	0/21/0	17/0/0	23/0/0
**Invasive mechanical ventilation (Yes/No/UD)**	0/21/0	1/18/0	17/3/1
**Diabetes (Yes/No/UD)**	1/20/0	4/13	2/20/1
**Dyslipidemia (Yes/No/UD)**	3/18/0	12/5	10/12/1
**Hypertension (Yes/No/UD)**	20/1	11/6	11/11/1
**DIC (Yes/No/UD)**	0/21	5/12	2/20/1
**Exitus (Yes/No/UD)**	21/0	0/17	7/16

DIC, Disseminated intravascular coagulation; ICU, Intensive Care Unit; IQR, Interquartile range; N/A, Not applicable; UD, Undetermined.

### Increased Levels of Neutralizing Antibodies Against SARS-CoV-2

**Figure 1:**
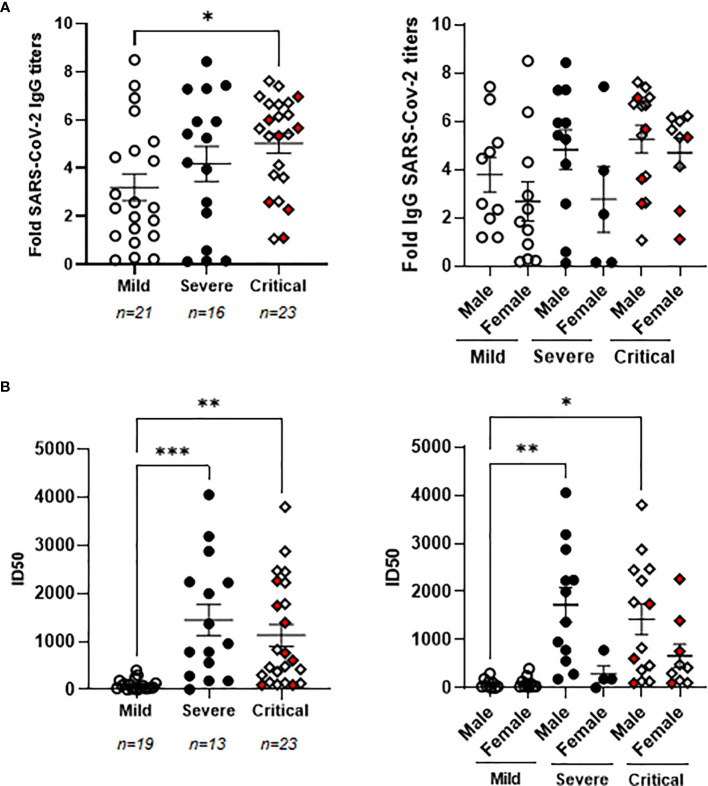
Characterization of the humoral response against SARS-CoV-2 in patients with different presentations of COVID-19. **(A)** Analysis of IgGs against SARS-CoV-2 spike protein in patients with mild, severe and critical COVID-19 recruited for this study (left graph) and divided by gender (right graph). **(B)** Titers of neutralizing antibodies calculated as ID50 in all patients with mild, severe and critical COVID-19 recruited for this study (left graph) and divided by gender (right graph). Each dot represents data from one individual. Red dots represent deceased patients. Statistical significance was calculated using one-way ANOVA and Tukey’s multiple comparisons test. *p < 0.05; **p < 0.01; ***p < 0.005.

Patients with critical COVID-19 showed levels of specific IgGs against SARS-CoV-2 that were increased 1.9-fold in comparison with patients with mild COVID-19 (*p*<0.05) (**Figure 1A**, left graph). There were no significant differences between the levels of these antibodies in the plasma of males and females (**Figure 1A**, right graph). Seven individuals from the cohort of critical COVID-19 deceased during their stay at the ICU (4 male and 3 female) but there was no significant change in the levels of specific IgGs against SARS-CoV-2 between the individuals with critical COVID-19 who deceased or survived at the ICU. The neutralizing capacity of these antibodies was analyzed using pNL4-3Δenv_SARS-CoV-2-SΔ19(G614)_Ren pseudovirus and ID50 was increased 13.7- and 11.3-fold in patients with severe (p<0.0001) and critical (p<0.01) COVID-19, respectively, in comparison with patients with mild COVID-19 (**Figure 1B**, left graph). The levels of neutralizing antibodies against SARS-CoV-2 were similar on average in critical patients who deceased in comparison with those with critical COVID-19 who survived. The titers of neutralizing antibodies were almost similar between men and women in mild patients, whereas they were 3.2- (p<0.05) and 2.6-fold higher in males than females, respectively, in plasma from severe and critical patients (**Figure 1B**, right graph). Accordingly, neutralizing antibodies were increased 18.0- (p<0.01) and 16.3-fold (p<0.01) in severe and critical males in comparison with males with mild COVID-19.

Levels of specific IgGs against SARS-CoV-2 correlated moderately with their neutralizing activity against the virus (Spearman r coefficient: 0.6065; 95% confidence interval (CI): 0.3952-0.7568; *p*<0.001).

### High Titers of IgGs Against Herpesviruses and Viral Reactivation in Plasma of Patients With Severe and Critical COVID-19

**Figure 2:**
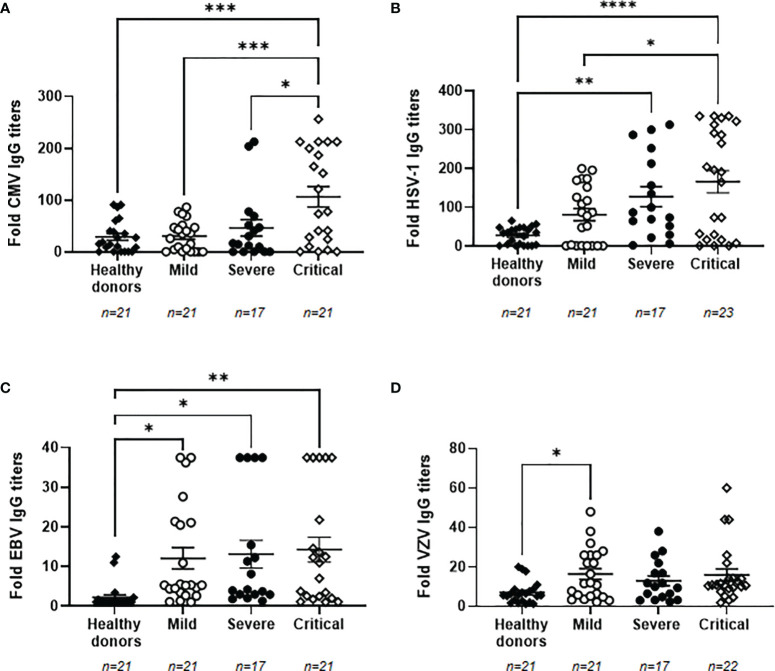
Levels of IgGs against prevalent herpesviruses in plasma from patients with different presentations of COVID-19. Levels of IgGs against CMV **(A)**, HSV-1 **(B)**, EBV **(C)** and VZV **(D)** in plasma from patients with mild, severe and critical forms of COVID-19. Data shown are mean ± SEM. Each dot represents data from one individual. Statistical significance was calculated using one-way ANOVA and Tukey’s multiple comparisons test. *p < 0.05; **p < 0.01; ***p < 0.001; ****p < 0.0001.

Due to the impaired immune response presented in individuals with the most severe forms of COVID-19 (7), the reactivation of latent herpesviruses was analyzed in our cohorts of patients. We determined that 100% of the individuals recruited for this study were seropositive for VZV. The percentage of seropositivity for EBV, CMV and HSV-1 was lower in healthy donors than in patients with COVID-19, being 40.9% and 100% for EBV, 68.2% and 80.65% for CMV, and 72.7% and 87.10% for HSV-1, respectively. Titers of specific IgGs against CMV were increased 3.6-fold in patients with critical COVID-19 in comparison with healthy donors (p<0.001) and patients with mild COVID-19 (p<0.001), whereas it was increased 1.6-fold in comparison with patients with severe COVID-19 (p<0.05) (**Figure 2A**). Specific IgG levels against HSV-1 also increased progressively in accordance to COVID-19 severity. They were increased 4.6- (p<0.01) and 6.0-fold (p<0.001) in individuals with severe and critical COVID-19, respectively, in comparison with healthy donors, and 2.0-fold in individuals with critical COVID-19 in comparison with those with mild disease (p<0.05) (**Figure 2B**) Specific IgG levels against EBV were increased 5.5- (p<0.05), 6.0- (p<0.05), and 6.5-fold (p<0.01) in patients with mild, severe, and critical COVID-19, respectively, in comparison with healthy donors (**Figure 2C**). A similar profile was observed for VZV because all patients with COVID-19 showed increased levels of specific IgGs against this herpesvirus. These IgGs were increased 2.3-fold in both groups of patients with mild and critical COVID-19 but this increment was only statistically significant in patients with mild COVID-19 (p<0.05), in comparison with healthy donors (**Figure 2D**). We did not find any significant correlation between the levels of specific IgGs against SARS-CoV-2 and all the herpesviruses that were analyzed.

**Figure 3:**
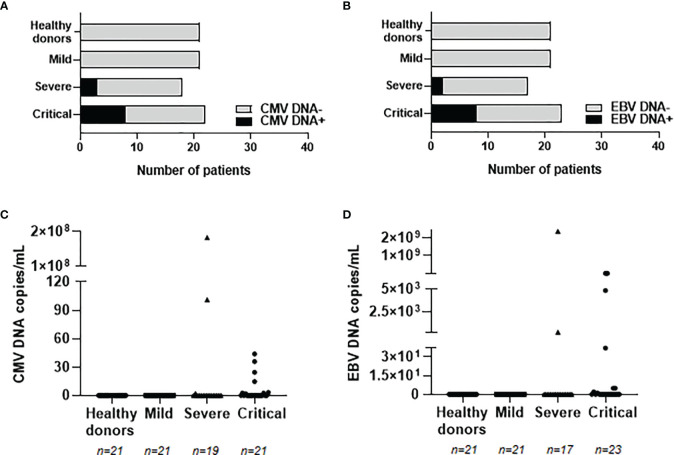
Detection and quantification of provirus reactivation from prevalent herpesviruses in plasma from patients with different presentations of COVID-19. Number of patients with proviral reactivation of CMV **(A)** or EBV **(B)** by specific qPCR. Copy number of CMV **(C)** and EBV **(D)** DNA per milliliter of plasma from patients with mild, severe and critical COVID-19. Each dot represents data from one individual.

In order to evaluate whether these enhanced levels of IgGs against herpesviruses were associated with viral reactivation from latency, the presence of viral DNA from CMV and EBV was analyzed in plasma from our cohorts of patients with COVID-19. The reactivation of HSV-1 and VZV was not analyzed due to the predominant tissue-localized infection (25, 26). CMV and EBV DNA was not detected in patients with mild COVID-19 or healthy donors, but it was detected in 17 hospitalized patients with severe or critical COVID-19 (see **Supplemental Table 2**). Median age of these patients was 66 years (IQR 42-90), 9 were males (53%) and 8 were females (47%). Eleven patients with severe (n=2) and critical (n=9) COVID-19 showed positive PCR for CMV DNA (**Figure 3A**). Similarly, ten patients with severe (n=2) and critical (n=8) COVID-19 showed positive PCR for EBV DNA (**Figure 3B**). Within these individuals, one patient with severe COVID-19 and three patients with critical COVID-19 showed viremia of both CMV and EBV. Copies per mL of reactivated proviruses greatly varied among the different patients, ranging from 2.20 to 182,866,000 copies/mL of CMV DNA (**Figure 3C**) and from 2.35 to 7,595,035 copies/mL of EBV DNA (**Figure 3D**). Decease due to COVID-19 was reported in three patients showing CMV or EBV positive viremia and in one patient with both CMV and EBV viremia, which accounts in total for 57.1% of the patients who died in our cohort. Relative risk ratio between reactivation of EBV and/or CMV and death during hospitalization due to COVID-19 was 0.8556, although it was not statistically significant.

### Changes in B Cell Subpopulations in Severe and Critical COVID-19 Patients

**Figure 4:**
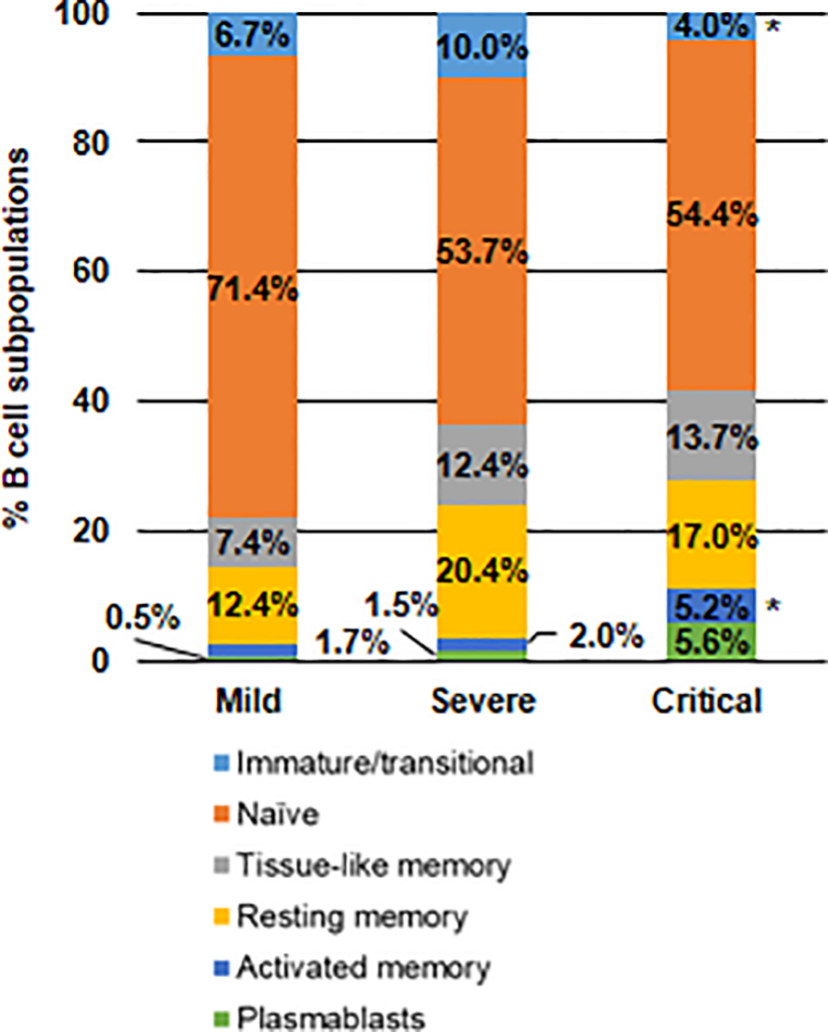
Analysis of the distribution of B cell subpopulations in patients with different presentations of COVID-19. Analysis by flow cytometry of B cell subpopulations in peripheral blood of patients with mild, severe and critical COVID-19 after staining with specific antibodies against markers CD10, CD127, CD20 and CD21. Statistical significance was calculated using one-way ANOVA and Tukey’s multiple comparisons test. *p < 0.05.

As COVID-19 increased in severity, it was observed a progressive enhancement of plasmablasts in peripheral blood, which were increased 3.7- and 11.2-fold, respectively, in severe and critical COVID-19, in comparison with mild COVID-19 (**Figure 4**). All memory B cell subpopulations were also increased in patients with severe and critical COVID-19, being the activated memory B cells those with a greater increase in comparison with mild COVID-19 (2.6- and 3.0-fold (p<0.05) more in patients with severe and critical COVID-19, respectively). This enhancement in activated B cell subpopulations induced 1.6- and 1.3-fold (p<0.05) decrease in naïve and immature/transitional B cells, respectively, in patients with critical COVID-19, in comparison with mild COVID-19.

### Impaired ADCC in PBMCs From Individuals With Severe and Critical COVID-19

**Figure 5:**
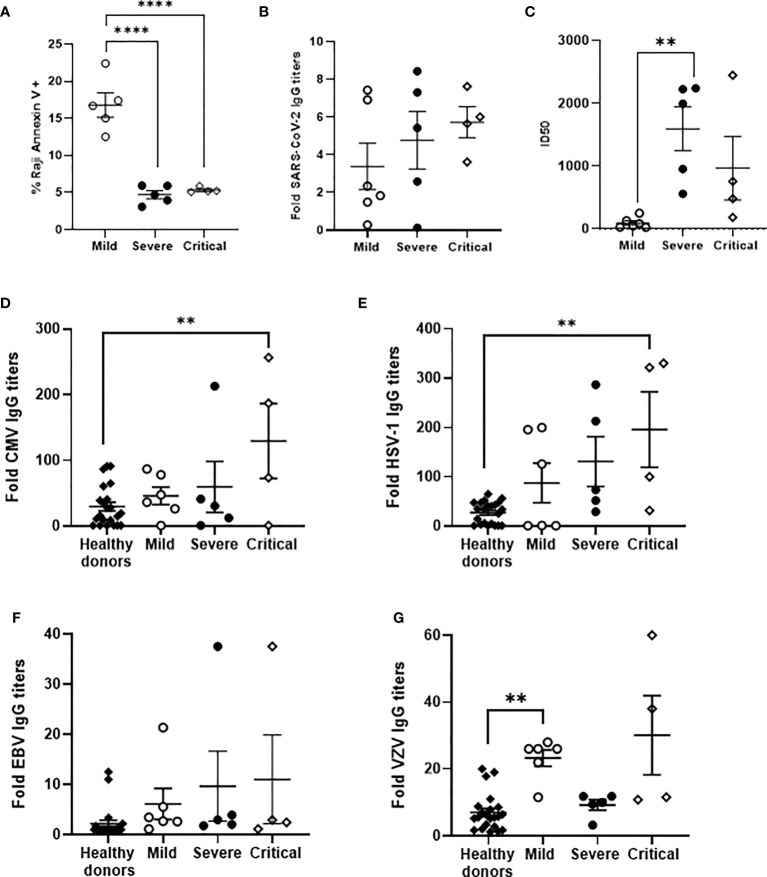
Analysis of ADCC activity in a subgroup of patients with different presentations of COVID-19 and their levels of IgGs against SARS-CoV-2 and prevalent herpesviruses. **(A)** Analysis of ADCC assay by quantification of Annexin V-PE as a measurement of apoptosis in PKH76-labelled, rituximab-coated Raji cells after co-culture with PBMCs (1:2) isolated from patients with mild, severe and critical COVID-19. Titers of total IgGs **(B)** and neutralizing antibodies, calculated as ID50 **(C)**, against SARS-CoV-2 spike protein in plasma from the same patients. Titers of IgGs against CMV **(D)**, HSV-1 **(E)**, EBV **(F)**, and VZV **(G)** in plasma from the same patients. Data shown are mean ± SEM. Statistical significance was calculated using one-way ANOVA and Tukey’s multiple comparisons test. **p < 0.01, ****p < 0.001.

ADCC was analyzed in a small subgroup of our cohort (see **Supplemental Tables 1** and **2**), according to sample availability. Median age was 73 in individuals with critical or severe COVID-19 and 41 years in individuals with mild COVID-19. Most hospitalized patients were male whereas individuals with mild COVID-19 were mostly female. None of these patients deceased during hospitalization and seven of nine hospitalized patients had hypertension as predominant risk factor. ADCC in PBMCs isolated from these individuals with severe and critical COVID-19, measured by the induction of apoptosis in rituximab-coated Raji cells, was reduced 3.2-fold (p<0.0001) in comparison with PBMCs from the patients with mild COVID-19 (**Figure 5A**). In this subgroup of individuals, the level of IgGs against SARS-CoV-2 were increased 1.4- and 1.7-fold in patients with severe and critical COVID-19 in comparison with mild patients (**Figure 5B**). The neutralizing capacity of these antibodies was increased 17.5- and 10.1-fold in patients with severe (p<0.01) and critical COVID-19, respectively, in comparison with patients with mild COVID-19 (**Figure 5C**).

Specific IgGs against CMV were increased 1.6-, 2.0- and 4.4-fold (p<0.01), respectively, in the plasma of these individuals with mild, severe and critical COVID-19 in which ADCC was analyzed, in comparison with healthy donors (**Figure 5D**). Similarly, the levels of IgGs against HSV were increased 3.2-, 4.8- and 7.1-fold (p<0.01), respectively, (**Figure 5E**); the levels of IgGs against EBV were increased 2.8-, 4.4- and 5.0-fold (**Figure 5F**), respectively; whereas the levels of IgGs against VZV were increased 3.3- (p<0.01) and 4.3-fold in patients with mild and critical COVID-19 but were similar to healthy donors in individuals with severe COVID-19 (**Figure 5G**). CMV DNA was detected in the plasma of two individuals with severe and critical COVID-19, whereas EBV DNA was also detected in two individuals with critical COVID-19. One of them showed reactivation of both CMV and EBV.

## Discussion

Clinical outcomes of COVID-19 vary from asymptomatic or mild disease to a severe and critical syndrome, suggesting the importance of the host immunity in the progression of the disease. Analysis of the humoral response in a Spanish cohort of patients with different presentations of COVID-19 showed that individuals with the most severe forms of the disease developed high titers of antibodies against SARS-CoV-2 in plasma, as was described in other studies (18, 34). These antibodies showed higher neutralizing capacity than those developed by individuals who had mild COVID-19 (15, 17–20). Similarly, higher titers of antibodies against SARS-CoV-2 with neutralizing capacity were found in some critical patients who deceased. Interestingly, we described here for the first time that the ability to neutralize SARS-CoV-2 was significantly increased in antibodies from plasma of hospitalized males than in females. In fact, most hospitalized patients in our cohort were males, according to previous reports in which the most critical forms of COVID-19 occur within this group of population (35).

The neutralizing activity of antibodies has been associated with protection against SARS-CoV-2 in animals models (36). Paradoxically, these antibodies were mostly detected in patients with the most severe presentations of COVID-19, which suggests that the immune response triggered by antibodies may be ineffectual in these individuals. The neutralizing antibodies have the ability to inhibit the viral dissemination through the organism but they need to be present at the time of the infection in order to induce the maximum protection. However, this may only happen in vaccinated individuals or those who had been previously in contact with the virus, which was not the case in our cohort of patients. The neutralizing antibodies may stop the viral dissemination once they have been developed, which usually occurs within 2-3 weeks after infection with SARS-CoV-2 (34, 37). Until then, the viral dissemination has to be stopped by other immune mechanisms. In this regard, ADCC activity mediated by NK cells and non-neutralizing IgG antibodies against SARS-CoV-2 spike glycoprotein also contributes to contain the viral spread though the elimination of the infected cells (22, 38). In fact, it has been demonstrated that both neutralizing capacity and additional functions of antibodies such as ADCC are needed to enhanced SARS-CoV-2 control and clearance (39). Despite the high IgG titers, ADCC activity was impaired in individuals from our cohort who showed severe or critical forms of COVID-19, which would result in a deficient SARS-CoV-2 elimination and would contribute to COVID-19 severity. Therefore, the high levels of antibodies in individuals with COVID-19 admitted to the ICU may be just a consequence of the high antigen exposure (21) but not an indication that an efficient immune response is being developed. Accordingly, it is critically important that both neutralizing and non- neutralizing antibody functions such as ADCC are evaluated during the test of efficacy of vaccines against SARS-CoV-2 infection (21), in addition to determine their applicability as biomarkers for disease severity and progression. Furthermore, the analytical characterization of SARS-CoV-2 antibodies generated by the different individuals, as well as their ability to trigger an effective cytotoxic response, could be needed to further understand the differential progression of COVID-19 due to the composition of N-glycans and polymorphic allotype variations in the Ig gamma heavy chain gene may modify the binding affinity and also, the ADCC response (40, 41). To our knowledge, this is the first time that impaired ADCC response has been described in patients with COVID-19 admitted to the ICU and it supports our previous observation that the cellular immune response against SARS-CoV-2 is compromised in these individuals with the most severe presentations of the disease (7).

We performed ADCC assays based on the antibody-dependent target cell lysis mediated by FcγRIIIa receptor (CD16), mostly expressed by NK cells. ADCC *via* NK cells against SARS-CoV-2 spike glycoprotein has been observed previously in plasma from recovered or recovering COVID-19 patients (22). However, NK cells from patients with severe COVID-19 show reduced levels and a dysfunctional, altered expression of receptors (7, 42, 43). As a result, impaired NK functions, including ADCC response, are surely essential to get a better understanding in COVID-19 poor progression. Other CD16-expressing cells, such as CD8γδ+ T cells, monocytes/macrophages, neutrophils and eosinophils may also trigger ADCC (44, 45). However, patients with severe outcomes of COVID-19 also show dysregulated immune responses associated to these cells. In fact, eosinopenia has been associated with high mortality due to COVID-19 (46, 47) and we have shown that highly cytotoxic CD8γδ+ T cell levels are greatly diminished in critical patients (7). The low levels of essential cells involved in ADCC activity may at least partly explain the impaired cytotoxicity observed in the PBMCs of the individuals from our cohort.

Durable humoral immunity against SARS-CoV-2 after recovery has been observed in individuals with any form of COVID-19 (14). However, antibody titers may also diminish and become undetectable after the resolution of the infection (17, 48, 49). This may be a consequence of a decline in the short-lived antibody-secreting B cells (9, 14). However, decrease in detectable levels of antibodies do not necessarily indicate that all acquired immunity will be lost because memory B cells may rapidly proliferate and differentiate into antibody-producing plasma cells in response to the antigen re-challenge, providing a fast and effective response even with undetectable levels of serum antibodies (14, 50). In this regard, the level of SARS-CoV-2-specific memory B cells increases steadily during the first 4-5 months after the infection and then stabilizes (15). Accordingly, we observed an increase of plasmablasts in the peripheral blood of patients from our cohort that were admitted to the ICU, as previously described (13, 14). The highest levels of plasmablasts may be detected in peripheral blood within the first 20 days after COVID-19 onset and then decline to baseline levels (9, 14). However, plasmablasts may remain in blood of patients with acute infection for longer periods, even after 2 months of the disease onset (9). In fact, although the median number of days from the clinical onset to sampling in patients from our cohort with the most critical form of COVID-19 was 42 days, the level of plasmablasts was still increased more than 2-fold in comparison with patients with severe COVID-19 that did not meet criteria for admission to the ICU. These results support the notion that in patients with the most severe forms of COVID-19 there is a sustained, acute immune response that cannot clear the infection successfully.

Other B cell memory phenotypes such as tissue-like memory, resting memory and activate memory B cells were also increased in the blood of individuals with severe and critical COVID-19, which correlated with the high antibody titers in plasma (14, 51). Non-antigen specific memory B cells may also expand during acute COVID-19 (52), which supports that the increased levels of memory B cells detected in our patients with critical COVID-19 reflected a prolonged acute phase. Furthermore, these memory B cells may even be related to other pathogens that are present in coinfection with SARS-CoV-2. In fact, the activation of a potent and sustained immune response during a viral infection may induce the reactivation of latent proviruses in patients with COVID-19 such as the herpesviruses CMV and EBV (24–28). In accordance, the levels of IgGs against the most prevalent herpesviruses CMV, HSV-1, EBV, and VZV were significantly increased in the plasma of patients with critical COVID-19 and reactivation of CMV and EBV proviruses was also detected in the blood of 25% of these patients, which correlated with fatal outcomes. EBV is a universal virus and about 90% of adults throughout the world have developed antibodies against it (53). We observed that IgG titers against EBV were quite low in our cohort of healthy donors in comparison with individuals with COVID-19 but this might be coincidental or maybe it could reflect an absence of viral reactivation in healthy donors. Besides, although IgGs against EBV capsid (VCA) usually persist for life, the antibody titers greatly fluctuate within the individuals and may not correlate with disease severity (54, 55). Co-infection of CMV or EBV with SARS-CoV-2 has been described previously in patients with COVID-19 (24–28) and it may contribute to the fatal outcome due to several reasons, such as the additional burden for the immune system and the potential immunosuppressive effect of some herpesviruses such as CMV (56, 57). Accordingly, we and others have described that immune cells from patients with COVID-19 show significantly higher levels of exhaustion markers such as PD-1 and Tim-3 (7, 43, 58), increased regulatory T cell (Treg) counts, CD4 lymphopenia, and impaired direct cytotoxicity (DCC) (7, 59, 60), which may consequently influence on the reactivation of CMV and EBV proviruses. Moreover, CMV reactivation has been associated with high levels of proinflammatory cytokines in plasma of the most critical COVID-19 patients (5), as well as with longer ICU stay. In accordance, our data showed that EBV and/or CMV reactivation probably contributed to the worst prognosis of COVID-19 in hospitalized patients. Additionally, CMV and EBV viremia has been described to correlate with the expansion of virus-specific memory B cells (61, 62). Therefore, we cannot rule out the possibility that EBV- and/or CMV-specific B cells may be contributing to the altered distribution of B cell subpopulations in patients with critical COVID-19 who showed herpesvirus reactivation in plasma.

NK cells are essential against CMV and/or EBV infections (63). Clonal expansion of activated NKG2C+ NK cells with enhanced ADCC activity has been described during CMV infection (63–65). However, the presence of SARS-CoV-2 appeared to alter ADCC response so potently that it may even overcome the CMV-induced ADCC activity. Therefore, the impaired ADCC response observed in patients with critical COVID-19 may account for CMV and EBV reactivation despite that activating receptors such as NKp44 and NKp46, involved in the killing of HSV-1-infected cells (66), and NKG2C, involved in the generation of CMV induced adaptive responses (67, 68), are generally overexpressed in these individuals (7).

In conclusion, individuals with the most severe forms of COVID-19 that were admitted to the ICU were mostly males and they developed a potent humoral response, as was demonstrated by increased populations of activated B cells in blood with capacity to synthesize high levels of neutralizing antibodies. However, this enhanced immune response was not translated into an efficient cytotoxic response with the ability to clear the infected cells *via* ADCC. Due to sample limitations, we could not include more samples for the analysis of ADCC, but nevertheless, the statistical significance was very high between groups. Other limitation of our study is that the number of days from clinical onset to sampling was different from hospitalized to non-hospitalized patients due to the official lockdown decreed by the Spanish Government during the first peak of COVID-19 pandemic, which prevented the homebound patients with mild COVID-19 to participate in any study until the confinement ended. Although a durable immune response against SARS-CoV-2 has been demonstrated 8 months after recovering from the infection (15), we may not rule out that this difference may have influenced the humoral response observed in patients with mild COVID-19. However, we did observe significant differences in the humoral response between patients with severe COVID-19 who were not admitted to the ICU, and those who stayed at the ICU with invasive mechanical ventilation. This is in accordance with the sustained immune response developed in patients with critical COVID-19. Besides, patients with the most severe forms of COVID-19 also showed reactivation of latent herpesviruses from previous infections, which could contribute to the poor prognosis of patients admitted to the ICU. In summary, this work is in line with the global efforts currently ongoing to get a better understanding about the immune response developed during SARS-CoV-2 infection, as well as to determine biomarkers of bad prognosis that may help to prevent the most severe outcomes of COVID-19.

## Data Availability Statement

The original contributions presented in the study are included in the article/**Supplementary Material**. Further inquiries can be directed to the corresponding authors.

## Ethics Statement

Hospitalized patients were recruited from Hospital Universitario Ramón y Cajal, Hospital Universitario Puerta de Hierro, and Hospital de El Escorial (Madrid, Spain). Non-hospitalized patients were recruited from Primary Healthcare Center Doctor Pedro Lain Entralgo (Madrid, Spain). Protocol for this study (CEI PI 32_2020-v2) were performed in accordance with the Helsinki Declaration and they were approved by the Ethics Committees of Instituto de Salud Carlos III (IRB IORG0006384) and all participating centers. The studies involving human participants were reviewed and approved by Comité de É tica Instituto de Salud Carlos III. The patients/participants provided their written informed consent to participate in this study.

## Contributing Members of the Multidisciplinary Group of Study of COVID-19 (MGS-COVID) (in Alphabetical Order)

Esther Alonso Herrador^1^, Pablo Amich Alemany^1^, Cristina Ávila Calzada^2^, Pilar Balfagón^7^, Victoria Bosch Martos^1^, Gema Carrillo Blanco^2^, Sandra Chamorro^3^, Belén Comeche^4^, Lorena Cordova Castaño^1^, Magdalena Corona de Lapuerta^3^, Susana Domínguez-Mateos^1^, Aurora Expósito Mora^1^, Valle Falcones^5^, Jesús de la Fuente^7^, Mario García Peña^2^, María Mercedes Gea Martinez^1^, Marta Gómez-Álvarez Domínguez^2^, Alberto Gomez Bonilla^1^, Lourdes Hernández^7^, María Victoria Leon Gomez^1^, Gema Lora Rey^1^, Alejandro Luna de Abia^3^, Patricia Mínguez^5^, Maria Luisa Muñoz Balsa^1^, María Ángeles Murillo^7^, Raquel Paz Peño^2^, Isabel Pérez^7^, Javier Pérez Gonzalez^1^, Sandra Pérez-Santos^1^, Daniel Renuncio García^2^, Adolfo J Saez-Marín^3^, Jose Sanchez Hernández^1^, Cruz Soriano^6^, Mercedes Toral Leiva^2^, Andrea Vinssac Rayado^1^

^1^Centro de Salud Doctor Pedro Laín Entralgo, Alcorcón, Spain

^2^Internal Medicine Service, Hospital Universitario Severo Ochoa, Leganés, Spain

^3^Hematology Service, Hospital Universitario Ramón y Cajal, Madrid, Spain

^4^Infectious Diseases Service, Hospital Universitario Ramón y Cajal, Madrid, Spain

^5^Neumology Service, Hospital Universitario Puerta de Hierro, Majadahonda, Spain

^6^Intensive Medicine Service, Hospital Universitario Ramón y Cajal, Madrid, Spain

^7^Serology Laboratory, National Center of Microbiology, Instituto de Salud Carlos III, Majadahonda, Spain

## Author Contributions

LV, MCo, and ML-H conceptualized the project and wrote the manuscript. LV, SR, MT, and EM processed and stored all blood samples. MCe, RM, CN, MM-A, and VG-G identified, selected, and recruited the patients, with the invaluable collaboration of all member of MGS-COVID. JG-P, VP, and JA conceptualized and performed the neutralization assays. MP-O and MCa determined the IgGs titers. SRM and MT performed the qPCR assays. LV, SR, MT, MCo, and ML-H collected and analyzed the clinical data and laboratory results. All authors contributed to the article and approved the submitted version.

## Funding

This work was supported by the Coordinated Research Activities at the Centro Nacional de Microbiología (CNM, Instituto de Salud Carlos III) (COV20_00679) to promote an integrated response against SARS-CoV-2 in Spain (Spanish Ministry of Science and Innovation) that is coordinated by Dr Inmaculada Casas (WHO National Influenza Center of the CNM) and a generous donation provided by Chiesi España, S.A.U. (Barcelona, Spain). The funder was not involved in the study design, collection, analysis, interpretation of data, the writing of this article or the decision to submit it for publication. This work was also supported by the Spanish Ministry of Economy and Competitiveness (PID2019 110275RB-I00); the Spanish AIDS Research Network RD16CIII/0002/0001 that is included in Acción Estratégica en Salud, Plan Nacional de Investigación Científica, Desarrollo e Innovación Tecnológica 2016-2020, Instituto de Salud Carlos III, European Region Development Fund (ERDF); Miguel Servet - AESI, MPY 341/21. The work of ML-H and SR is financed by NIH grant R01AI143567. The work of MT is supported by Instituto de Salud Carlos III (COV20_00679). The work of LV is supported by a predoctoral grant from Instituto de Salud Carlos III (FIS PI16CIII/00034-ISCIII-FEDER).

## Conflict of Interest

The authors declare that the research was conducted in the absence of any commercial or financial relationships that could be construed as a potential conflict of interest.

## Publisher’s Note

All claims expressed in this article are solely those of the authors and do not necessarily represent those of their affiliated organizations, or those of the publisher, the editors and the reviewers. Any product that may be evaluated in this article, or claim that may be made by its manufacturer, is not guaranteed or endorsed by the publisher.
