# Supplementary figures and images for: Impaired Antibody-Dependent Cellular Cytotoxicity in a Spanish Cohort of Patients With COVID-19 Admitted to the ICU
Source: Front Immunol. 2021 Sep 20;12:742631. doi: 10.3389/fimmu.2021.742631 (PMC8488389; doi:10.3389/fimmu.2021.742631)

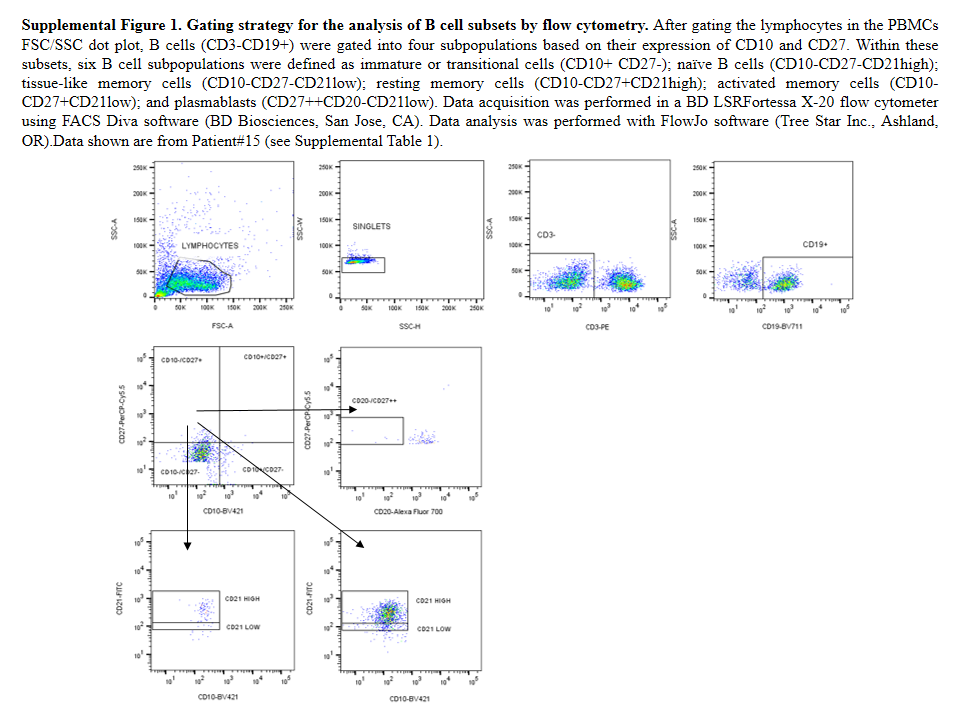

Supplement: Supplementary file 1 [file Image_1.tif]
