# Supplementary material for: Impaired Antibody-Dependent Cellular Cytotoxicity in a Spanish Cohort of Patients With COVID-19 Admitted to the ICU
Source: Front Immunol. 2021 Sep 20;12:742631. doi: 10.3389/fimmu.2021.742631 (PMC8488389; doi:10.3389/fimmu.2021.742631)
Supplement: Supplementary file 2 [file Table_1.docx]

**Supplemental Table 1.** Clinical characteristics of non-hospitalized patients with COVID-19 who were recruited for this study at the Primary Healthcare Center Doctor Pedro Laín Entralgo (Madrid, Spain).

| **Patient's ID** | **Age (years)** | **Gender** | **Days from clinical onset to sample** | **Exitus** | **Cough and expectoration** | **Dyspnea** | **Fever** | **Pneumonia** | **Bilateral pneumonia** | **Diarrhea and vomiting** | **Lethargy** | **Migraine** | **Asthenia** | **Treatment** | **DM** | **DL** | **HT** |
| --- | --- | --- | --- | --- | --- | --- | --- | --- | --- | --- | --- | --- | --- | --- | --- | --- | --- |
| **1** | 64 | M | 75 | No | Yes | Yes | Und | No | No | Und | No | No | Yes | HCQ | Yes | Yes | No |
| **2** | 57 | F | 85 | No | Yes | Yes | Yes | Yes | Und | Yes | No | No | Yes | HCQ | No | No | No |
| **3** | 58 | F | 73 | No | No | No | No | No | No | No | No | No | Yes | NA | No | No | No |
| **4** | 50 | F | 81 | No | Yes | No | Yes | No | No | No | No | No | Yes | NA | No | No | No |
| **5**^#^ | 53 | F | 87 | No | Yes | No | Und | No | No | No | No | No | Yes | NA | No | No | No |
| **6**^#^ | 41 | F | 83 | No | Yes | No | Yes | No | No | No | No | Yes | Yes | NA | No | No | No |
| **7** | 29 | M | 87 | No | No | No | Yes | No | No | No | No | No | Yes | NA | No | No | No |
| **8**^#^ | Und | M | Und | No | No | No | Und | No | No | No | No | No | No | NA | No | No | No |
| **9**^#^ | 26 | F | 87 | No | Yes | Yes | Yes | No | No | No | No | No | Yes | LMWH | No | No | No |
| **10** | 26 | M | 88 | No | No | No | No | No | No | Yes | No | No | Yes | NA | No | No | No |
| **11** | 28 | M | 79 | No | Yes | No | Yes | No | No | No | No | No | Yes | NA | No | No | No |
| **12** | 31 | M | 90 | No | No | Yes | Und | Yes | No | Yes | No | No | Yes | NA | No | No | No |
| **13** | 62 | M | 82 | No | Yes | Yes | Yes | No | No | No | No | No | Yes | HCQ | No | No | No |
| **14** | 52 | F | 72 | No | Yes | No | Yes | Und | Und | No | Und | No | Yes | NA | No | No | No |
| **15** | 57 | F | 66 | No | No | No | Yes | No | Und | Yes | Und | Yes | Yes | NA | No | Yes | No |
| **16** | 29 | F | 83 | No | Yes | Yes | No | No | No | No | No | No | Yes | NA | No | No | No |
| **17** | 45 | M | 82 | No | Yes | No | Yes | No | No | No | No | Yes | Yes | NA | No | Yes | No |
| **18** | 27 | M | 85 | No | No | No | Yes | No | No | Yes | No | No | Yes | NA | No | No | No |
| **19**^#^ | 26 | F | 74 | No | No | No | Yes | Und | Und | No | No | No | No | NA | No | No | No |
| **20** | 32 | M | 95 | No | Yes | No | Yes | Und | Und | Yes | No | Yes | No | NA | No | No | No |
| **21**^#^ | 47 | F | 99 | No | Yes | Yes | Yes | No | No | Yes | Yes | Yes | Yes | HCQ | No | No | Yes |

M: male; F: female; Und: undetermined; NA: not applicable; HCQ: hydroxychloroquine; LMWH: Low-molecular-weight heparin; DM: Diabetes mellitus, DL: dyslipidemia; HT: hypertension. ^#^ Antibody dependent cellular cytotoxicity (ADCC) was analyzed.
