# Supplementary material for: Impaired Antibody-Dependent Cellular Cytotoxicity in a Spanish Cohort of Patients With COVID-19 Admitted to the ICU
Source: Front Immunol. 2021 Sep 20;12:742631. doi: 10.3389/fimmu.2021.742631 (PMC8488389; doi:10.3389/fimmu.2021.742631)
Supplement: Supplementary file 3 [file Table_2.docx]

**Supplemental Table 2.** Clinical characteristics of hospitalized patients with severe and critical COVID-19 recruited for the study.

| **Patient's ID** | **COVID-19** | **Age (years)** | **Gender** | **Days from clinical onset to sample** | **Exitus** | **Cough and expectoration** | **Dyspnea** | **Fever** | **Bilateral pneumonia** | **Diarrhea and vomiting** | **Lethargy** | **Migraine** | **Asthenia** | **Treatment** | **DM** | **DL** | **HT** | **DIC** | **CVM / EBV viremia** |
| --- | --- | --- | --- | --- | --- | --- | --- | --- | --- | --- | --- | --- | --- | --- | --- | --- | --- | --- | --- |
| 22 | Severe | 66 | M | Und | NO | Und | Und | YES | YES | Und | Und | Und | Und | HCQ, LPV/r, AZM, CS | NO | NO | NO | NO | EBV |
| 23 | Severe | 77 | M | 35 | NO | NO | YES | YES | YES | NO | NO | NO | YES | HCQ, LPV/r, AZM, TOZ, CS | YES | YES | YES | NO | NO |
| 24 | Severe | 63 | F | 14 | NO | Und | YES | NO | YES | YES | NO | NO | YES | HCQ, LPV/r, AZM, CS | NO | NO | YES | NO | NO |
| 25^#^ | Severe | 72 | M | 28 | NO | YES | NO | YES | YES | NO | NO | NO | YES | HCQ, AZM, CS | NO | NO | YES | NO | NO |
| 26^#^ | Severe | 72 | M | 40 | NO | YES | NO | YES | YES | NO | NO | NO | NO | HCQ , LPV/r, TOZ, CS | YES | YES | YES | YES | NO |
| 27 | Severe | 76 | M | 25 | Und | YES | NO | NO | YES | NO | NO | NO | NO | HCQ , LPV/r, AZM, LMWH | NO | YES | YES | NO | NO |
| 28 | Severe | 72 | M | 10 | NO | NO | NO | YES | YES | NO | NO | NO | NO | HCQ , LPV/r, AZM, LMWH, TOZ, CS | NO | YES | NO | YES | NO |
| 29 | Severe | 67 | F | 18 | NO | NO | NO | YES | YES | NO | NO | NO | YES | HCQ, LPV/r, AZM,LMW, TOZ, CS | NO | NO | NO | NO | CMV |
| 30^#^ | Severe | 80 | M | Und | NO | Und | YES | Und | YES | Und | YES | Und | Und | HCQ, AZM, LMWH, CS | YES | NO | YES | YES | NO |
| 31 | Severe | 59 | F | 32 | NO | YES | YES | YES | YES | NO | NO | NO | NO | HCQ, AZM, | YES | YES | NO | NO | NO |
| 32 | Severe | 72 | F | 1 | NO | YES | YES | NO | YES | YES | NO | NO | NO | HCQ, AZM, TOZ, CS | NO | NO | NO | NO | NO |
| 33^#^ | Severe | 73 | M | 42 | NO | YES | NO | NO | YES | NO | NO | NO | NO | LPV/r, AZM, TOZ, CS | NO | NO | YES | NO | NO |
| 34 | Severe | 84 | F | 52 | NO | YES | NO | NO | YES | NO | NO | YES | NO | HCQ, LPV/r, AZM, CS | NO | NO | YES | NO | NO |
| 35 | Severe | 69 | M | 17 | NO | NO | NO | YES | YES | NO | NO | NO | NO | HCQ, TOZ, CS | NO | NO | YES | YES | NO |
| 36^#^ | Severe | 89 | M | Und | NO | NO | NO | NO | YES | YES | NO | NO | NO | HCQ | NO | NO | YES | YES | CMV |
| 37 | Severe | 50 | M | 3 | NO | YES | NO | NO | YES | NO | NO | NO | NO | HCQ | NO | NO | YES | NO | NO |
| 38 | Severe | 90 | M | Und | NO | YES | YES | NO | YES | NO | NO | NO | NO | HCQ, AZM, CS | NO | NO | NO | NO | EBV/CMV |
| 39 | Critical | 58 | M | 43 | NO | YES | YES | U | YES | NO | NO | NO | YES | HCQ, LPV/r, AZM, TOZ, CS | NO | YES | YES | YES | NO |
| 40 | Critical | 67 | M | 23 | YES | NO | YES | YES | YES | NO | NO | NO | NO | HCQ, AZM, TOZ, CS | NO | YES | YES | NO | NO |
| 41 | Critical | 63 | F | 28 | NO | YES | YES | YES | YES | NO | NO | NO | NO | HCQ,TOZ, CS | NO | YES | NO | YES | NO |
| 42 | Critical | 42 | M | 39 | NO | YES | YES | YES | YES | NO | NO | NO | YES | HCQ, LPV/r, AZM, LMWH, TOZ, CS | NO | NO | YES | Und | EBV/CMV |
| 43 | Critical | 66 | F | U | NO | NO | NO | YES | YES | NO | YES | NO | NO | Und | NO | YES | YES | NO | NO |
| 44 | Critical | 69 | M | 1 | YES | Und | Und | Und | YES | Und | Und | Und | Und | Und | Und | Und | Un | NO | NO |
| 45 | Critical | 59 | M | 44 | NO | YES | YES | YES | YES | NO | NO | YES | NO | HCQ, LPV/r, AZM, LMWH, TOZ, CS | NO | NO | YES | NO | EBV |
| 46 | Critical | 50 | M | 48 | NO | YES | NO | NO | YES | NO | YES | YES | NO | HCQ, LPV/r, AZM, LMWH, CS | NO | NO | YES | NO | NO |
| 47 | Critical | 56 | M | 39 | NO | YES | YES | YES | YES | NO | NO | YES | NO | HCQ, LPV/r, AZM, LMWH, TOZ, CS | NO | NO | YES | NO | NO |
| 48 | Critical | 72 | F | 55 | NO | NO | YES | YES | YES | NO | NO | NO | NO | HCQ , LPV/r, AZM, LMWH, CS | NO | NO | YES | NO | CMV |
| 49 | Critical | 58 | F | 38 | NO | YES | YES | YES | YES | YES | NO | YES | YES | HCQ , LPV/r, AZM, LMWH, TOZ, CS | NO | NO | NO | NO | EBV |
| 50 | Critical | 56 | F | 18 | NO | YES | NO | NO | YES | NO | NO | NO | NO | HCQ , LPV/r, LMWH, CS | NO | NO | NO | NO | CMV |
| 51 | Critical | 69 | M | 55 | NO | YES | YES | YES | YES | NO | NO | YES | NO | HCQ , LPV/r, AZM, LMWH, TOZ, CS | NO | NO | NO | NO | CMV |
| 52 | Critical | 62 | M | 50 | YES | YES | YES | YES | YES | NO | NO | YES | NO | HCQ , LPV/r, AZM, TOZ, CS | NO | YES | NO | NO | NO |
| 53 | Critical | 64 | M | 53 | YES | YES | YES | YES | YES | NO | NO | NO | NO | HCQ , LPV/r, AZM, LMWH, TOZ, CS | YES | YES | NO | NO | CMV |
| 54 | Critical | 63 | F | 46 | YES | NO | NO | NO | YES | NO | NO | NO | NO | HCQ, LMWH, CS | NO | NO | NO | NO | EBV |
| 55 | Critical | 50 | M | 14 | NO | YES | YES | YES | YES | NO | NO | YES | NO | HCQ , LPV/r, AZM, LMWH, CS | NO | NO | NO | NO | NO |
| 56^#^ | Critical | 72 | F | 59 | NO | YES | YES | YES | YES | NO | NO | YES | NO | HCQ , LPV/r, AZM, LMWH, TOZ, CS | NO | YES | YES | NO | NO |
| 57^#^ | Critical | 73 | M | 19 | NO | YES | YES | YES | YES | NO | NO | NO | NO | HCQ , LPV/r, AZM,LMW, TOZ, CS | NO | YES | NO | NO | CMV |
| 58^#^ | Critical | 65 | M | 25 | NO | YES | YES | YES | YES | NO | NO | YES | NO | HCQ , LPV/r, AZM, LMWH, TOZ, CS | NO | NO | NO | NO | EBV/CMV |
| 59^#^ | Critical | 73 | F | Und | NO | NO | NO | NO | YES | NO | NO | NO | NO | HCQ , LPV/r, AZM, LMWH, TOZ, CS | NO | NO | YES | NO | EBV |
| 60 | Critical | 79 | F | 27 | YES | YES | YES | YES | YES | NO | NO | NO | NO | CS; TOZ | NO | YES | NO | NO | EBV |
| 61 | Critical | 88 | M | 7 | YES | NO | YES | NO | YES | NO | NO | NO | NO | NO | YES | YES | YES | NO | EBV/CMV |

M: male; F: female; Und: Undetermined; NA: not applicable; HCQ: hydroxychloroquine; LPV/r: lopinavir/ritonavir; AZM: azithromycin; LMWH: Low-molecular-weight heparin; TOZ: tocilizumab; CS: corticosteroids; DIC: Disseminated Intravascular Coagulation; DM: Diabetes mellitus, DL: dyslipidemia; HT: hypertension; EBV: Epstein-Barr virus; CMV: cytomegalovirus.

^#^Antibody dependent cellular cytotoxicity (ADCC) was analyzed.

**Supplemental Table 2 (continuation).** Data collected during hospitalization of patients with severe and critical COVID-19 who were recruited for this study.

| **Patient's ID** | **COVID-19** | **Hospitalization** | | | | | | | **Blood biochemistry data** | | | | | | | | | | |
| --- | --- | --- | --- | --- | --- | --- | --- | --- | --- | --- | --- | --- | --- | --- | --- | --- | --- | --- | --- |
|  |  | **Hospitalization time at sampling (days)** | **Hospitalization time since sampling (days)** | **Days in ICU** | **NIV** | **IV** | **Reservoir** | **Nasal Glasses** | **CRP (mg/mL)** | **LDH (U/L)** | **CK (U/L)** | **Ferritin (ng/mL)** | **IL-6 (pg/mL)** | **D-dimer (ug/mL)** | **Procalcitonin (ng/mL)** | **Lymphocytes (cells/uL)** | **Monocytes (cells/uL)** | **Platelets (cells/uL)** | **Fibrinogen (mg/dL)** |
| 22 | Severe | 23 | 23 | NA | NO | NO | NO | YES | 118.8 | 478 | 3337 | 1045 | 8.7 | 2.0 | Und | 300 | 200 | 80000 | 516 |
| 23 | Severe | 67 | 31 | NA | YES | NO | YES | YES | 125.1 | 732 | 33 | 1334 | 767.9 | 33.9 | 0.08 | 400 | 200 | 82000 | 166 |
| 24 | Severe | 8 | 2 | NA | NO | NO | NO | NO | 104.9 | 3.04 | 61 | 222 | Und | 3.1 | Und | 2850 | 700 | 262000 | 358 |
| 25^#^ | Severe | 24 | 22 | NA | NO | NO | NO | NO | 146.3 | Und | 51 | 4821 | Und | 0.6 | 0.15 | 500 | 500 | 116000 | 819 |
| 26^#^ | Severe | 50 | 35 | NA | YES | NO | YES | YES | 363.5 | 632 | 102/11 | 1033 | 4624 | 10.1 | 1 | 800 | 100 | 190000 | 778 |
| 27 | Severe | 21 | 23 | NA | NO | NO | NO | YES | 178.4 | 364 | 248 | 1544 | 144.1 | 2.0 | Und | 400 | 1200 | 144000 | 998 |
| 28 | Severe | 40 | 35 | NA | NO | NO | YES | NO | 234.8 | 1454 | 93 | 2462 | 1497.8 | 2.7 | 0.05 | 25.8 | 0.3 | 117000 | 785 |
| 29 | Severe | 29 | 17 | NA | YES | NO | NO | YES | 155.5 | 707 | 13 | 622 | 3718.3 | 4.8 | 0.2 | 1000 | 200 | 128000 | 710 |
| 30^#^ | Severe | 38 | 20 | NA | NO | NO | NO | YES | 156.6 | 449 | 18 | 2374 | Und | 9.9 | Und | 700 | 3200 | 97000 | 694000 |
| 31 | Severe | 10 | 7 | NA | YES | NO | NO | YES | 157 | 581.8 | 37 | 29 | Und | 0.4 | 10 | 4100 | 15000 | 47000 | 570 |
| 32 | Severe | 9 | 25 | NA | YES | NO | NO | YES | 78.2 | 596 | 192 | 620 | 12.3 | 1.0 | 0.37 | 300 | 1100 | 480000 | 573 |
| 33^#^ | Severe | 38 | 32 | NA | NO | NO | NO | YES | 173 | 489 | 273 | 0.6 | 5855.66 | 3.1 | 0.6 | 390 | Und | 70000 | 195 |
| 34 | Severe | 44 | 42 | NA | YES | YES | YES | YES | 152 | 878 | 338 | 0.31 | Und | 6.8 | 0.31 | 190 | Und | 106000 | 447 |
| 35 | Severe | 16 | 11 | NA | YES | NO | NO | YES | 23.4 | 495 | 13 | 2356 | Und | 0.5 | 2.64 | 460 | 1180 | 79000 | U |
| 36^#^ | Severe | 32 | 15 | NA | NO | NO | NO | NO | 29.19 | 463 | 7012 | 1719 | Und | 8.6 | Und | 460 | 1590 | 79000 | U |
| 37 | Severe | 10 | 5 | NA | NO | NO | NO | NO | 113 | 217 | 134 | 0.03 | Und | 1.1 | 0.03 | 190 | 10 | 214000 | 418 |
| 38 | Severe | 13 | 12 | NA | NO | NO | NO | NO | 13.52 | 202 | 7 | 1557 | Und | 1.4 | 0.11 | 380 | 40 | 119000 | U |
| 39 | Critical | 45 | 39 | 13 | YES | YES | NO | YES | 187.8 | 627 | 2043 | 1101 | 233.6 | 13.1 | Und | 300 | 600 | 247000 | 174 |
| 40 | Critical | 29 | 19 | NA | YES | NO | NO | NO | 115.6 | 1230 | 240 | 4535 | 2232 | 3.0 | 0.33 | 200 | 300 | 151000 | 814 |
| 41 | Critical | 34 | 21 | NA | NO | NO | NO | NO | 250 | 801 | 561 | 2688 | U | 8.6 | Und | 800 | 1200 | 130 | 915 |
| 42 | Critical | 34 | 22 | 26 | YES | YES | NO | YES | 112.9 | 374 | 384 | 563 | U | 8.9 | 0.29 | 200 | 200 | 705000 | 703 |
| 43 | Critical | 13 | 30 | 10 | YES | NO | NO | YES | 209.5 | 668 | 13-258 | 999 | 74.2 | 5.5 | 0.06 | 600 | 0 | 652-11 | 799 |
| 44 | Critical | 38 | 19 | Und | Und | Und | Und | Und | 88.9 | 1068 | 139 | 2166 | 960.1 | 3.4 | U | 400 | 100 | 71000 | 590 |
| 45 | Critical | 45 | 43 | 7 | NO | YES | YES | YES | 360 | 561 | 72 | 9.34 |  | 4.0 | 9.34 | 340 | 40 | 166000 | 309 |
| 46 | Critical | 52 | 44 | 13 | NO | YES | YES | YES | 326 | 1096 | 535 | 0.55 | 41.4 | 6.7 | 0.55 | 440 | 260 | 150000 | 519 |
| 47 | Critical | 37 | 35 | 30 | NO | YES | YES | YES | 36.8 | 590 | 309 | 0.55 | 134.96 | 5.3 | 0.55 | 210 | 250 | 102000 | 422 |
| 48 | Critical | 94 | 53 | 61 | NO | YES | YES | YES | 273.6 | 880 | 347 | 4.34 | 39.85 | 3.2 | 4.34 | 210 | 140 | 12000 | 407 |
| 49 | Critical | 96 | 31 | 42 | NO | YES | YES | YES | 276 | 971 | 3517 | 0.72 | 524.8 | 35.0 | 0.72 | 250 | 250 | 55600 | 130 |
| 50 | Critical | 81 | 45 | 37 | NO | YES | YES | YES | 231 | 635 | 85 | 2.31 | 24.88 | 35.0 | 2.31 | 410 | 0 | 138000 | 400 |
| 51 | Critical | 110 | 48 | 76 | NO | YES | YES | YES | 304 | 548 | 236 | 0.66 | 163.09 | 8.9 | 0.66 | 240 | 230 | 87000 | 414 |
| 52 | Critical | 103 | 48 | 94 | NO | YES | YES | YES | 491 | 726 | 236 | 1.78 | 696.49 | 9.5 | 1.78 | 130 | 110 | 48300 | 100 |
| 53 | Critical | 54 | 52 | 56 | YES | YES | YES | YES | 188 | 120 | 197 | 0.39 | 499 | 8.8 | 0.39 | 380 | 260 | 186000 | 120 |
| 54 | Critical | 26 | 15 | 54 | NO | NO | NO | YES | 374 | 900 | 528 | 3.41 | 273.79 | 11.5 | 3.41 | 170 | 50 | 157000 | 277 |
| 55 | Critical | 23 | 8 | 13 | NO | YES | YES | YES | 339 | 576 | 238 | 1.68 | 385 | 5.1 | 1.68 | 350 | 140 | 223000 | 340 |
| 56^#^ | Critical | 92 | 57 | 66 | NO | YES | YES | YES | 311 | 629 | 374 | 11.73 | 1240 | 6.0 | 11.73 | 320 | 240 | 177000 | 217 |
| 57^#^ | Critical | 36 | 13 | 49 | YES | YES | YES | YES | 104 | 774 | 192 | 0.39 | 476 | 6.1 | 0.39 | 490 | 360 | 80000 | 112 |
| 58^#^ | Critical | 105 | 51 | 90 | NO | YES | YES | YES | 309 | 785 | 869 | 0.35 | 21 | 11.7 | 0.35 | 550 | 150 | 72000 | 243 |
| 59^#^ | Critical | 45 | 39 | 16 | NO | YES | YES | YES | 317 | 584 | 112 | 0.21 | 1057 | U | 0.21 | 490 | 140 | 142000 | 445 |
| 60 | Critical | 14 | 14 | 14 | YES | NO | NO | NO | 22.8 | 864 | NA | NA | NA | 1000 | 0.15 | 990 | 710 | 295000 | 740 |
| 61 | Critical | 2 | 2 | NA | NO | NO | YES | NO | 289.9 | 1006 | 678 | NA | NA | 34244 | 1.29 | 1670 | 1320 | 232000 | 740 |

ICU: Intensive Care Unit; NIV: Non-invasive mechanical ventilation; IV: Invasive mechanical ventilation; CRP: C Reactive Protein; LDH: lactate dehydrogenase; CK: Creatine kinase; IL-6: Interleukin-6; Und: Undetermined.
